# Presence of glucose, xylose, and glycerol fermenting bacteria in the deep biosphere of the former Homestake gold mine, South Dakota

**DOI:** 10.3389/fmicb.2013.00018

**Published:** 2013-02-15

**Authors:** Gurdeep Rastogi, Raghu N. Gurram, Aditya Bhalla, Ramon Gonzalez, Kenneth M. Bischoff, Stephen R. Hughes, Sudhir Kumar, Rajesh K. Sani

**Affiliations:** ^1^Department of Chemical and Biological Engineering, South Dakota School of Mines and TechnologyRapid City, SD, USA; ^2^Departments of Chemical and Biomolecular Engineering and Bioengineering, Rice UniversityHouston, TX, USA; ^3^Renewable Product Technology Research Unit, Agricultural Research Service, National Center for Agricultural Utilization Research, US Department of AgriculturePeoria, IL, USA

**Keywords:** bioenergy, bioethanol, biofuels, fermentation, gold mine, 1,3-propanediol

## Abstract

Eight fermentative bacterial strains were isolated from mixed enrichment cultures of a composite soil sample collected at 1.34 km depth from the former Homestake gold mine in Lead, SD, USA. Phylogenetic analysis of their 16S rRNA gene sequences revealed that these isolates were affiliated with the phylum Firmicutes belonging to genera *Bacillus* and *Clostridium.* Batch fermentation studies demonstrated that isolates had the ability to ferment glucose, xylose, or glycerol to industrially valuable products such as ethanol and 1,3-propanediol (PDO). Ethanol was detected as the major fermentation end product in glucose-fermenting cultures at pH 10 with yields of 0.205–0.304 g of ethanol/g of glucose. While a xylose-fermenting strain yielded 0.189 g of ethanol/g of xylose and 0.585 g of acetic acid/g of xylose at the end of fermentation. At pH 7, glycerol-fermenting isolates produced PDO (0.323–0.458 g of PDO/g of glycerol) and ethanol (0.284–0.350 g of ethanol/g of glycerol) as major end products while acetic acid and succinic acid were identified as minor by-products in fermentation broths. These results suggest that the deep biosphere of the former Homestake gold mine harbors bacterial strains which could be used in bio-based production of ethanol and PDO.

## Introduction

Lignocellulosic biomass represents an inexpensive, abundant, and renewable biological resource that has great potential for the production of biofuels (Himmel et al., 2007). Many factors, such as the highly resistant nature of lignin and the crystallinity of cellulose along with structural complexity make lignocellulose recalcitrant toward biological and chemical degradation (Zheng et al., 2009). Alkaline pretreatment breaks lignin-carbohydrate bonds in lignocellulosic biomass and has been used extensively to increase the enzymatic digestibility of switchgrass and prairie cordgrass, which are promising biomass feedstocks especially in South Dakota (Gonzalez-Hernandez et al., 2009; Karunanithy and Muthukumarappan, 2011). However, the major problem associated with alkali pretreatment is that it raises the pH of medium containing biomass to the highly alkaline (>10) range, which is not optimal for fermenting microorganisms such as *Saccharomyces cerevisiae* and *Zymomonas mobilis* that perform fermentation only under acidic pH (5.0) conditions (Zaldivar et al., 2001; Zheng et al., 2009). At industrial scale of biomass fermentation, large pH shifts (highly alkaline) require significant use of acids, resulting in an increased amount of waste salt products that must be disposed of appropriately. Furthermore, *S. cerevisiae* and *Z. mobilis* are of limited use in fermentation of biomass with high pentose content, as they cannot ferment pentose sugars unless they are genetically engineered to express the desired pathways (Zaldivar et al., 2001). Although, ethanol producing strains of *Escherichia coli* can degrade pentose and hexose sugars, the fermentation reactions are generally carried out at pH 7. Therefore, *E. coli* is also not suitable for fermentation of alkali-treated biomass (Zaldivar et al., 2001). From this perspective, novel bacteria with different spectra of abilities, such as those that can ferment glucose and xylose sugars under alkaline conditions, will lead to a cost-effective and environmentally friendly bioethanol production process.

Another problem associated with bioconversion of feedstocks such as soybean, vegetable, canola, and waste oils and animal fats into biofuels is the generation of glycerol as a major by-product (Yazdani and González, 2007; Yazdani et al., 2010). For example, it has been shown that for every 100 kg of biodiesel or bioethanol produced, the amounts of crude glycerol produced are 4 and 10 kg, respectively (Barbirato et al., 1998; Maervoet et al., 2011). With increased emphasis placed on bioethanol and biodiesel production, there is also an increase in the amount of crude glycerol produced. In Europe, the amount of waste glycerol produced was more than 700 kilotons in year 2008. In the US, the amount of crude glycerol produced has reached approximately 251 kilotons per year (Valdivieso, 2010). Crude glycerol contains several impurities (alcohol, salts, and heavy metals) and for small-scale biofuels industries, purification of this crude glycerol is not economical. As more and more crude glycerol is produced from the biofuel industries, methods to use this waste product effectively will be advantageous to further minimize the cost of production of biofuels. One such method is microbial transformation of waste glycerol to valuable products such as 1,3-propanediol (PDO) which offers a promising alternative to manage waste glycerol.

PDO is a very valuable chemical that is used in the synthesis of various polyesters, polyurethane, lubricant, solvent, and as a precursor in the chemical and pharmaceutical industries (Saxena et al., 2009; Maervoet et al., 2011). Although conventional chemical methods do exist for the synthesis of PDO, the process is quite expensive due to use of high pressure, temperature, and expensive catalysts along with generation of toxic intermediates. Thus, anaerobic microbial conversion of crude glycerol to produce PDO is an area of extensive research and provides a way to capitalize on the surplus of waste glycerol (Thompson and He, 2006). However, so far only a few bacterial species belonging to genera *Clostridium, Klebsiella, Enterobacter, Lactobacillus, Citrobacter*, and *Bacillus* have been shown to convert glycerol into PDO under anaerobic conditions (Zhao et al., 2006; Willke and Vorlop, 2008; da Silva et al., 2009; Saxena et al., 2009). Considering the rapidly growing market for PDO and to deal with the problem of waste glycerol, there is increasing need to isolate and characterize new strains of bacteria that can ferment glycerol into PDO.

It is apparent that there are several constraints associated with biofuels production such as limited availability of bacterial strains that can ferment alkali-treated biomass or ferment glycerol to commodity chemicals (e.g., ethanol, PDO). Considering these limitations, we have isolated glucose-, xylose-, or glycerol-fermenting bacteria from the extreme deep biosphere environment of the former Homestake gold mine, Lead, SD. There are very few reports on subsurface isolates utilizing glucose for ethanol production (Alain et al., 2002; Slobodkin et al., 2003; Slobodkina et al., 2008) but the information on xylose and glycerol fermenting deep subsurface microbes is relatively scarce. Deep subsurface microbes, *Tepidibacter thalassicus* and *Clostridium tepidiprofundi* sp. nov., fermented glucose into ethanol, acetate, and H_2_ (Slobodkin et al., 2003; Slobodkina et al., 2008). Another strain, *Caminicella sporogenes* gen. nov., sp. nov., produced H_2_, acetate, butyric acid and ethanol when grown on glucose as a substrate (Alain et al., 2002). In this study we characterized the biotechnological potential of seven strains for ethanol and PDO production using laboratory batch fermentation studies.

## Materials and methods

### Site description and soil collection

The Homestake mine (44°35′2074″N, 103°75′082″W; Lead, SD) is the deepest mine (2.4 km deep) in North America and had the largest gold deposits ever found in the Western Hemisphere (Rastogi et al., 2009b). This former gold mine is now known as Sanford Underground Research Facility (SURF). A detailed description of the mine is located at http://www.dusel.org/. In May 2008, a composite soil sample was collected at a depth of 1.34 km from the Ross shaft as described earlier (Rastogi et al., 2010a,b). A schematic cross section, location of sampling site, and elemental composition of the soil samples used in the present study have been described elsewhere (Rastogi et al., 2009b).

### Enrichment and isolation of glucose-, xylose-, and glycerol-fermenting bacteria

The glucose and xylose fermentation medium contained (per liter): 0.1 g nitriloacetic acid, 1 ml FeCl_3_ solution (0.03%), 0.05 g CaCl_2_·2H_2_O, 0.1 g MgSO_4_·7H_2_O, 0.005 g methionine, 1.8 g of 85% H_3_PO_4_, 0.05 g yeast extract, 0.01 g casamino acids, 0.01 g KCl, 0.3 g NH_4_Cl, and 1 ml of Nitsch's trace element solution (Rastogi et al., 2009a). The pH of the medium was adjusted to 10 using 10 M NaOH/glycine mixture to specifically enrich alkaliphilic fermentative bacteria. After autoclaving, the medium was supplemented with filter-sterilized solutions of D-xylose or D-glucose as a source of carbon to achieve a final concentration of 5 g/L of these sugars. For enrichment and isolation of glycerol-fermenting bacteria, the medium contained the following components (per liter): 7 g K_2_HPO_4_, 2 g KH_2_PO_4_, 2 g NH_4_Cl, 2 g MgSO_4_.7H_2_O, 0.5 g NaCl, 6.61 g (NH_4_)_2_SO_4_, 40 g glycerol, and 1 g yeast extract, and the pH of the medium was adjusted to 7. The glycerol fermentation medium was derived from the study of Zheng et al. (2008).

One gram of soil sample was aseptically added to 125-ml serum bottles containing 100 ml of pre-sterilized xylose, glucose, or glycerol fermentation medium. The serum bottles were sealed with butyl rubber stoppers, crimped with aluminum seals, and sparged with ultra-pure N_2(g)_ for 15 min to remove the dissolved and head space oxygen for creating anaerobic conditions (Sani et al., 2010). All enrichments were performed by incubating the serum bottles at 37°C in an incubator shaker (120 rpm) for 2–4 days. Triplicate serum bottles were used for each enrichment experiment, and controls included were: (1) soil samples autoclaved at 121°C, (2) soil-free controls, and (3) carbon source-free controls. Growth of enrichment cultures was monitored periodically by measuring total cell protein using a quantitative colorimetric Coomassie assay (Sani et al., 2001) and cultures showing growth were transferred into fresh fermentation medium. This process was repeated five times prior to initiating the isolation of pure cultures by deep agar plate technique from mixed enrichments. In brief, 100 μl of fifth-generation mixed enrichment cultures was added to 100 ml of sterile and lukewarm xylose, glucose, or glycerol agar medium, mixed well and poured immediately into petri plates. Triplicate agar plates from each enrichment culture were incubated under anaerobic conditions in BBL® Gas-Pak® containers (Becton Dickinson) for 2–4 days in the dark at 37°C. After incubation well-separated colonies that were embedded into agar were picked and inoculated into serum bottles containing fresh medium having a particular carbon source (glucose, xylose, or glycerol).

### 16S rRNA gene sequence analysis of glucose-, xylose-, and glycerol-fermenting bacteria

Total DNA was extracted from 10 ml of pure cultures of glucose-, xylose-, and glycerol-fermenting isolates. PCR amplification of nearly full-length 16S rRNA genes from each isolate was carried out using the primer set 8f/1492r and amplification conditions as described earlier (Rastogi et al., 2009a). Sequencing of 16S rRNA genes was performed commercially, and similarity searches for sequences were performed by BLAST (N). Sequences were aligned using the CLUSTALW, and phylogenetic trees were constructed using the neighbor-joining method (1000, bootstrap replicates) by MEGA v 3.1 (Kumar et al., 1993). All 16S rRNA sequences generated in this study have been deposited in GenBank under accession numbers GQ254068–GQ254079.

### Measurement of total cell protein, metabolites, and substrate consumption

The isolates were grown anaerobically in serum bottles containing 100 ml liquid medium with a particular carbon source xylose, glucose or glycerol. After the exponential phase of growth, the cells from seed cultures were re-inoculated into serum bottles containing 100 ml medium with the same carbon source, and anaerobic conditions were created. The inoculated bottles were incubated at 37°C under shaking conditions (120 rpm) for 6 days. Samples were withdrawn periodically and analyzed for soluble intermediates and end products. Samples were prepared for high-pressure liquid chromatography (HPLC) by centrifuging 1-ml cultures at 10,000 rpm, and the resulting supernatant was further filtered using 0.2 μm pore size membrane filters (Gelman Acrodisc). HPLC employed a 300 mm Aminex HPX 87H column (Bio-Rad Laboratories, Inc., Hercules, CA) on a HP 1100 Series HPLC system equipped with a refractive index detector (Agilent Technologies, Santa Clara, CA). Samples (10 μl) were injected onto a heated column (65°C) and eluted at 0.6 ml/min using 5 mM H_2_SO_4_ as mobile phase. The identification of fermentation product PDO was confirmed using HPLC and 1D proton nuclear magnetic resonance as described earlier (Dharmadi and Gonzalez, 2005; Murarka et al., 2008). All experiments were performed in duplicate and control serum bottles with no carbon source were also included.

### Determination of specific growth rates and yield coefficients

Bacterial growth rate is a time-dependent variable in cultures. In each sample discrete growth rate measurements were calculated for each time step. Equation 1 was used to obtain each discrete growth rate datum point as described earlier (Chhatwal, 2008).

(1) $$\mu_{i}=\left(\frac{1}{t}\right)ln\left(\frac{X_{i}}{X_{0}}\right)$$

In Equation 1 “μ_*i*_” represents the calculated growth rate (in hr^−1^) and “*X*_*i*_” is the measured cell mass concentration (g/L). The “*i*” subscript is a counter variable for each time step. Similarly, “*X*_0_” represents cell concentration at time *t* = 0. The variable “*t*” is time (hour). The arithmetic mean of data obtained was then calculated and represented as specific growth rate, “μ”.

Yield coefficients (g of biomass [X] or product [P] formed per g of substrate [S] consumed) were defined based on the amount of consumption of xylose, glucose, or glycerol. Growth yields were calculated from Equation 2.

(2) $$Y_{X/S}\equiv -\frac{\Delta X}{\Delta S}$$

Product yields on substrate were calculated from Equation 3.

(3) $$Y_{P/S}=-\frac{\Delta P}{\Delta S}$$

## Results and discussion

### Molecular identification of glucose-, xylose-, and glycerol-fermenting bacteria

**Figure 1:**
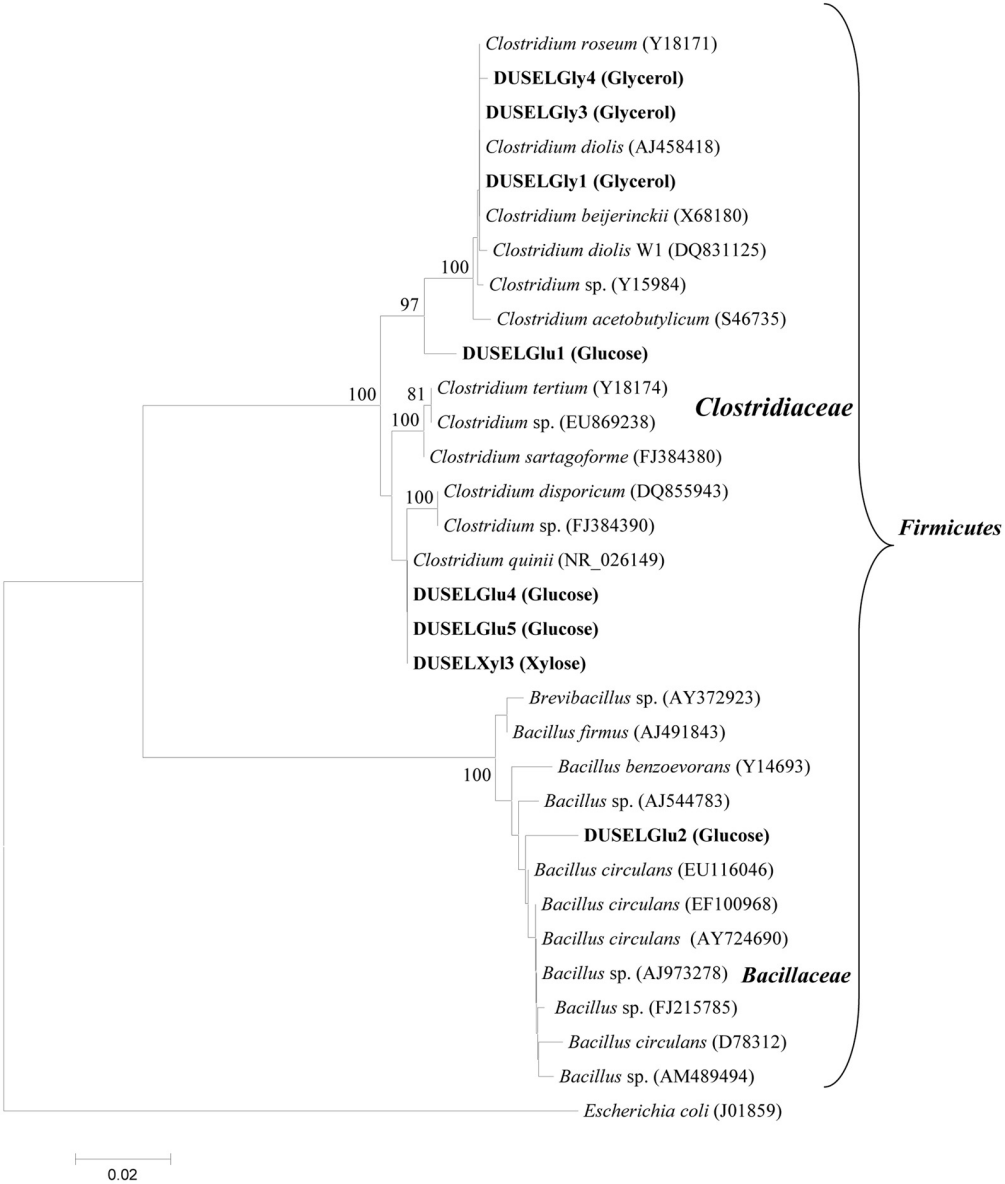
**Phylogenetic tree showing the relationship of 16S rRNA gene sequences retrieved from xylose-, glucose-, or glycerol-fermenting DUSEL strains with reference sequences of *Clostridium* and *Bacillus* sp. obtained from GenBank.** The type of enrichment medium from which a particular strain was isolated has been indicated in parenthesis along with the strain name in bold. *E. coli* (J01859) was selected as out-group to root the tree. The scale bar represents 0.02 substitutions per nucleotide position. Bootstrap values which were <75% are not shown.

A total of eight bacterial strains were purified from three different enrichment cultures and were used to ferment glucose, xylose, or glycerol. Figure 1 shows the phylogenetic identification of these isolates and the type of enrichment culture from which they have been purified. All isolates were found affiliated with phylum *Firmicutes* and grouped within the *Clostridiaceae* and *Bacillaceae* families. A majority of isolates (7 out of 8) were closely related to *Clostridium* sp. except a single isolate (DUSELGlu2) that closely grouped with sequences from *Bacillus* sp. These findings were in agreement with earlier studies from the Homestake mine, where *Clostridium* sp. has been shown to be the most predominant member (up to 62.3%) followed by *Bacillus* sp. (32.7%) in cellulose-degrading enrichment cultures (Rastogi et al., 2009a). *Clostridium* spp. have been shown to ferment glucose, xylose, or glycerol to produce various alcohols (e.g., ethanol, butanol), acids (e.g., lactic, acetic, butyric acids), and PDO (Balasubramanian et al., 2001; Ni and Sun, 2009; Jiang et al., 2010; Maervoet et al., 2011). Although several *Bacillus* species have been reported to produce ethanol (Romero et al., 2007; Ou et al., 2009), none of them has been shown to ferment glucose into ethanol especially under alkaline pH conditions as observed in the case of the DUSELGlu2 strain that belonged to genus *Bacillus* based on 16S rRNA phylogenetic analysis (Figure 1).

### Batch fermentation of xylose by deep-mine isolate

**Figure 2:**
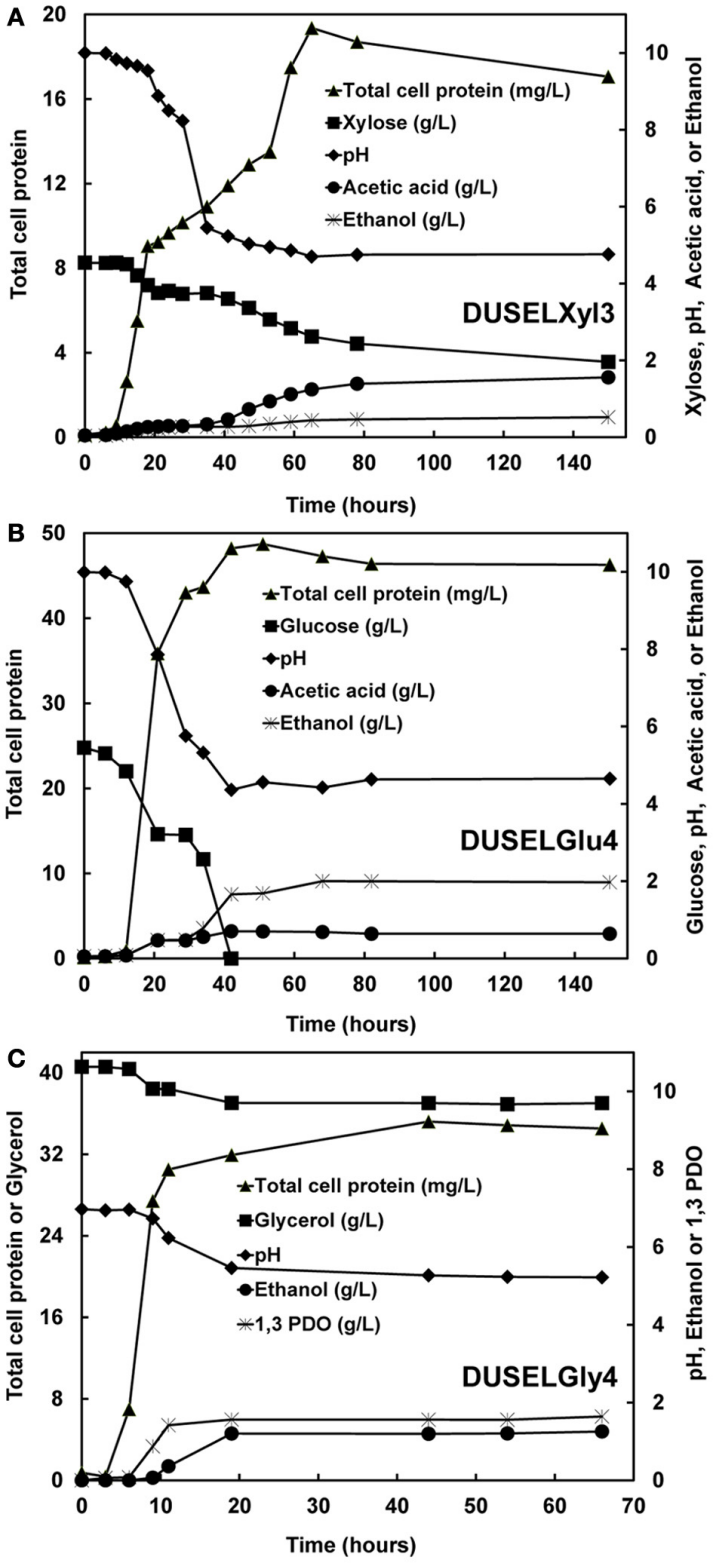
**Profiles of total cell protein, substrate consumption, change in pH, and product formation during the fermentation by DUSELXyl3 (A), DUSELGlu4 (B), and DUSELGly4 (C)**.

Xylose fermentation by DUSELXyl3 strain was studied in anaerobic batch cultures. After a lag of 6 h, DUSELXyl3 strain started utilizing xylose as carbon source, which was evident by a net increase in total cell protein and a decrease in xylose concentration in fermentation broth (Figure 2A). During the exponential growth phase, xylose fermentation produced acetic acid that decreased the pH of fermentation broth from 10 to 4.5 (Figure 2A). It is possible that low pH conditions generated in the fermentation broth might have affected metabolic pathways involved in xylose utilization and probably inhibited them. This was in line with batch fermentation profile of DUSELXyl3 strain which revealed this strain fermented only about 43% of the initial xylose concentration during 150 h of incubation (Figure 2A). HPLC analysis revealed that DUSELXyl3 produced acetic acid (1.56 g/L, data not shown) as a primary metabolite rather than ethanol (0.52 g/L) at the end of fermentation. DUSELXyl3 produced 0.189 g of ethanol/g of xylose which was about 37% of theoretical yield for complete conversion of sugar to ethanol.

### Batch fermentation of glucose by deep-mine isolates

Glucose-fermenting strains DUSELGlu1, DUSELGlu2, and DUSELGlu4 demonstrated similar batch fermentation profiles with respect to product formation, substrate consumption, and pH shifts in fermentation broth. For example, within 40 h of fermentation, DUSELGlu4 completely utilized the glucose that was available in the broth indicating that glucose can be readily fermented into ethanol in a short span of time. In all three glucose-fermenting strains, ethanol (0.205–0.304 g ethanol/g glucose) was detected as the primary product at the end of fermentation with small amounts of acetic acid (0.593–0.742 g/l, data not shown), and lactic acid (0.013–0.45 g/L, data not shown). Interestingly, the concentration of lactic acid dropped below the limit of detection at the end of fermentation in DUSELGlu1 and DUSELGlu4 strains, while in DUSELGlu2 strain, it turned out to be the third fermentation product along with ethanol and acetic acid. All three glucose-fermenting isolates showed similar ethanol yields and specific growth rates (Tables 1 and 2). Based on a theoretical maximum yield of 0.51 g ethanol/g sugars (Krishnan et al., 1999), the yield of ethanol in DUSELGlu2 strain was 0.304 g of ethanol/g of glucose which was 60% of theoretical maximum yield.

**Table 1:** **Growth rates and yield coefficients of xylose-, glucose-, and glycerol-fermenting deep-mine isolates**.

**Isolate**	**DUSELXyl3**	**DUSELGlu1**	**DUSELGlu2**	**DUSELGlu4**	**DUSELGly1**	**DUSELGly3**	**DUSELGly4**
Substrate used in fermentation	Xylose	Glucose	Glucose	Glucose	Glycerol	Glycerol	Glycerol
Specific growth rate μ(h^−1^)	0.025	0.030	0.032	0.038	0.031	0.037	0.048
Growth yield^a^ (*Y*_*X/S*_)	0.006	0.006	0.007	0.008	0.007	0.007	0.007
pH (initial − final)	10.0 − 4.50	10.0 − 4.76	10.0 − 3.7	10.0 − 4.65	7.0 − 5.22	7.0 − 5.32	7.0 − 5.22

Calculations were done as described in “Materials and Methods” under “Determination of Specific Growth Rates and Yield Coefficients” section.

a Growth yield (Y_X/S_)—g of biomass produced per g of substrate (xylose, glucose, or glycerol) consumed.

**Table 2:** **Products and yield coefficients of xylose-, glucose-, or glycerol-fermenting deep-mine isolates**.

**Isolate**	**Substrate used in fermentation**	**Fermentation end products**		**Yield coefficients**	
		**Major**	**Minor**	**Ethanol yield**^**a**^ **(Y**_**ET/S**_**)**	**PDO yield**^**b**^ **(Y**_**PD/S**_**)**
DUSELXyl3	Xylose	Acetic acid and Ethanol	None	0.189	NA
DUSELGlu1	Glucose	Ethanol	Acetic acid	0.205	NA
DUSELGlu2	Glucose	Ethanol	Acetic acid and Lactic acid	0.304	NA
DUSELGlu4	Glucose	Ethanol	Acetic acid	0.293	NA
DUSELGly1	Glycerol	PDO and Ethanol	Acetic acid and succinic acid	0.284	0.323
DUSELGly3	Glycerol	PDO and Ethanol	Acetic acid and succinic acid	0.298	0.367
DUSELGly4	Glycerol	PDO and Ethanol	Acetic acid and succinic acid	0.350	0.458

Calculations were done as described in “Materials and Methods” under “Determination of Specific Growth Rates and Yield Coefficients” section.

a Ethanol yield (Y_ET/S_)—g of ethanol formed per g of substrate (xylose, glucose, or glycerol) consumed.

b PDO Yield (Y_PD/S_)—g of PDO formed per g of glycerol consumed.

NA—not applicable.

The specific growth and metabolite product profiles of both xylose- and glucose-fermenting deep-mine strains showed that ethanol was not a growth-associated metabolite as it was formed after the exponential phase of growth (Figures 2A and B for DUSELXyl3 and DUSELGlu4, respectively). In previous studies, strains of *E. coli, Klebsiella oxytoca*, and *Zymomonas mobilis* have been shown to produce very high amounts (23–63 g/L) of ethanol from xylose and glucose fermentations (Yomano et al., 1998; Dien et al., 2003). Noticeably, these studies have used mutant/recombinant strains over-expressing a desired metabolic pathway, specialized growth media, sugar mixtures, and inoculation protocols. Our data on the fermentation of glucose, xylose, and glycerol by deep-mine isolates were generated under un-optimized minimal medium and culture conditions using wild-type strains. Therefore, detailed comparisons of deep-mine strains with earlier reports were not possible. Nonetheless, recombinant *E. coli* strains have been shown to produce 0.5 g ethanol/g of glucose in LB medium (de Carvalho Lima et al., 2002). Noticeably, our wild-type DUSELGlu2 strain produced 0.304 g ethanol/g of glucose in a minimal fermentation medium (Table 2). Furthermore, metabolically engineered *S. cerevisiae* strains have been shown to produce 0.35–0.38 g ethanol/g of xylose (Eliasson et al., 2000).

### Batch fermentation of glycerol by deep-mine isolates

All three glycerol-fermenting isolates, DUSELGly1, DUSELGly3, and DUSELGly4, displayed similar growth, substrate consumption, and product formation characteristics. The growth profile was very rapid and reached an exponential phase within 10 h of fermentation (Figure 2C for DUSELGly4). Once the fermentation reached a stationary phase at 66 h, only about 10% of the initial glycerol concentration was consumed by these isolates leading to the formation of PDO (1.44–1.64 g/L) and ethanol (1.18–1.27 g/L) as the major fermentation products. Due to the formation of acidic products (e.g., acetic and succinic acid), the pH of the fermentation broth decreased rapidly from 7.0 to 5.2 during exponential growth phase. Unlike acetic acid which appeared as a byproduct (0.09 g/L) along with PDO and ethanol at the end of fermentation, the concentration of succinic acid dropped below the limit of detection at the end of batch fermentation (data not shown). Specific growth and metabolite product profiles suggested that PDO was a growth-associated product (concentration increased with increase in cell growth—Figure 2C). HPLC analysis of fermentation broths also detected two peaks which were not identified and further investigations of these peaks are needed to ensure the identities of all end products that were formed during glycerol fermentation. These peaks may represent 2-3, butanediol, butanol, or butyric acid as previous studies have shown such intermediates in the oxidative and reductive pathways for the fermentation of glycerol in major PDO producers such as *Clostridium, Klebsiella, Lactobacillus*, and *Citrobacter* (Biebl, 1991; Maervoet et al., 2011; Rodriguez et al., 2012).

Molecular analysis based on 16S rRNA sequence suggested that all glycerol-fermenting isolates were closely related to *Clostridium* sp. (Figure 1). Interestingly, bacterial strains belonging to genus *Clostridium* have been shown as the best “PDO producers” and extensively used in bio-based production of PDO because of their appreciable substrate tolerance, yield, and productivity (González-Pajuelo et al., 2006; Chatzifragkou et al., 2011; Wilkens et al., 2012). DUSELGly4 demonstrated a higher specific growth rate of 0.048 h^−1^, ethanol yield of 0.35 g/g, and PDO yield of 0.458 g/g in comparison with other two glycerol-fermenting deep-mine strains (Figure 2C, Tables 1 and 2). The PDO yield of DUSELGly4 (0.458 g PDO/g of glycerol) was comparable to other reported species such as *K. pneumoniae* M5al 0.41 g/g (Cheng et al., 2006) and 0.496 g/g *C. butyricum* (González-Pajuelo et al., 2004). Overall these results suggest that DUSELGly isolates characterized in this study can ferment glycerol to PDO at pH 7 with comparatively good yield.

## Conclusions

The former Homestake gold mine represents a promising source for bioprospecting high-value microbes and microbial enzymes including those capable of degrading lignocellulosic biomass. Fermentation capabilities of glucose- and xylose-fermenting strains at alkaline pH have application in ethanol production from alkali-treated biomass. In an earlier study, we have shown that soil samples that were used to enrich and isolate glucose-, xylose-, and glycerol-fermenting strains contained high amounts of toxic metals such as As, Cd, Cu, and Pb due to mining activities (Rastogi et al., 2009a,b). Although, we did not study glycerol fermentation in the presence of toxic metals, isolation of fermenting strains from soil with high levels of toxic metals indicates that these bacteria would be adapted to tolerate toxic heavy metals. These newly isolated deep-mine strains, if they have metal-tolerance, would be of particular biotechnological interest as heavy metals are generally present as impurities in crude glycerol (Johnson and Taconi, 2007). DUSEL strains also showed potential for the production of major industrial products including acetic acid (e.g., by DUSELXyl3) and PDO. The efficiency of these strains could be further improved by optimizing growth media composition and culture conditions. In addition, pH-controlled batch experiments should be run to show the capabilities of ethanol production by these mine isolates at high pHs. A better understanding of the inhibition effect of ethanol, PDO, and pH on growth rates of deep-mine isolates will be required to overcome any inhibitory effect that these might have on sugar fermentation.

Deep-mine strains will also be evaluated to test their ability to ferment all three carbon sources (xylose, glucose, and glycerol). Molecular characterization of deep-mine strains using techniques such as DNA–DNA hybridization or using other phylogenetic marker genes are also needed. Further elucidation of metabolic pathways leading to the formation of ethanol, acetic acid, or PDO will be desirable for the construction of recombinant strains for industrial applications.

### Conflict of interest statement

The authors declare that the research was conducted in the absence of any commercial or financial relationships that could be construed as a potential conflict of interest.
